# Sjögren’s syndrome complicated with hypothyroidism and osteomalacia: A case report

**DOI:** 10.1097/MD.0000000000039771

**Published:** 2024-09-27

**Authors:** Runtian Chen, Lihua Fang, Xiaokang Fang, Jie Ning

**Affiliations:** a Department of Endocrinology, Shenzhen Longhua District Central Hospital, Shenzhen, Guangdong, China.

**Keywords:** autoimmune disorder, hypokalemia, osteomalacia, renal tubular acidosis, Sjögren’s syndrome

## Abstract

**Rationale:**

Sjögren syndrome (SS) is a prevalent autoimmune disorder targeting exocrine glands, causing symptoms such as dry eyes and mouth. It often goes underdiagnosed due to its varied presentations, emphasizing the importance of early and accurate diagnosis.

**Patient concerns:**

A 22-year-old female presented with atypical symptoms of hypokalemic paralysis and severe bone pain, which are not commonly associated with SS.

**Diagnoses:**

Extensive diagnostic workup, including serological tests, ophthalmological assessments, and a lip biopsy, confirmed the diagnosis of distal renal tubular acidosis as a complication of SS.

**Interventions:**

The patient was treated with an intensive inpatient regimen designed to stabilize her potassium levels and alleviate her symptoms.

**Outcomes:**

The comprehensive therapeutic intervention was successful, with the patient’s symptoms being alleviated within 2 weeks.

**Lessons:**

This case underscores the importance of being aware of SS in younger demographics and the necessity for a prompt and multifaceted treatment approach to manage systemic effects and improve quality of life.

## 1. Introduction

Sjögren’s syndrome (SS) is a chronic autoimmune disorder characterized by lymphocytic infiltration of exocrine glands, leading to symptoms such as dry eyes and mouth.^[1]^ It is a relatively common condition, affecting between 1% and 3% of the general population, with a higher prevalence in women, particularly between the ages of 50 and 80 years.^[2]^ The disease is often underdiagnosed due to its diverse manifestations and the lack of specific diagnostic markers, but its recognition is crucial as it can involve multiple organ systems and has the potential to significantly impact quality of life.^[3]^ Despite the complexity of SS, recent advances in understanding its pathophysiology have led to the development of targeted therapies, including biologics, which hold promise for improving patient outcomes.

## 2. Case report

We report on a 22-year-old female patient who initially presented with hypokalemic paralysis and severe bone pain, indicative of osteomalacia. Her medical history included recurrent episodes of hypokalemia and muscle weakness following a period of hunger and exhaustion, which had been previously treated with potassium supplements. Despite these interventions, she continued to experience symptomatic hypokalemia over the subsequent 4 years. In 2018, she was diagnosed with hypothyroidism but had a brief course of thyroid hormone treatment due to adverse drug reactions. She reported no history of smoking, alcohol consumption, or recreational drug use, and had no family history of hypokalemic episodes. Notably, she complained of persistent left hip pain for the past 6 months.

Upon admission to Longhua District Central Hospital on January 6, 2019, her serum potassium level was measured at 3.09 mmol/L. Physical examination revealed a height of 164 cm, weight of 52 kg, body mass index of 19.3 kg/m², and blood pressure of 105/64 mm Hg. She exhibited signs of weakness and pain without clinical dry eyes or dry mouth. Cardiopulmonary evaluation was normal, with no organomegaly detected. Neurological examination showed reduced muscle strength in both the upper and lower extremities, with decreased tendon reflexes, yet intact sensory and vibratory functions.

Comprehensive diagnostic workup, including laboratory tests (as detailed in Table 1A), arterial blood gas analysis, and urine biochemistry, revealed hypokalemia, metabolic acidosis, hypophosphatemia, and hypocalcemia, consistent with a diagnosis of distal renal tubular acidosis (dRTA). Dual-energy X-ray absorptiometry confirmed reduced bone mass in her lumbar spine and femoral neck, correlating with her reported bone pain. The presence of chronic autoimmune thyroiditis and severe hypothyroidism was confirmed by low thyroid hormone levels and elevated thyroid-stimulating hormone levels. Autoimmune antibodies, including those against Sjögren’s syndrome antigens A and B, antinuclear antibodies, and rheumatoid factors, were positive, suggesting the diagnosis of Sjögren’s syndrome. Ophthalmological tests indicated decreased tear film stability and secretion, and a lip biopsy showed chronic atrophic salivary glands with significant lymphocytic and plasmatic cell infiltration, further supporting the diagnosis of SS. The integration of these findings led to the diagnosis of SS complicated by dRTA, hypothyroidism, and osteomalacia.

### 2.1. Diagnostic methods

Our patient underwent a comprehensive laboratory investigation, the results of which are detailed in Table 1A. Key findings included hypokalemia at 3.09 mmol/L, anion gap metabolic acidosis with bicarbonate (HCO3‐) at 15.5 mmol/L and anion gap at 20.4 mmol/L, hypophosphatemia at 0.62 mmol/L, and hypocalcemia at 2.06 mmol/L. Arterial blood gas analysis revealed a pH of 7.33, a partial pressure of carbon dioxide (pCO2) of 28.2 mm Hg, and a partial pressure of oxygen (pO2) of 133 mm Hg. Urine biochemistry showed a specific gravity of 1.009 and a pH of 7.0. Consistent alkaline urine (pH range 6.5–7.5) was observed on repeated testing, with no signs of proteinuria, glucosuria, or aminoaciduria. Notably, serum creatinine levels were within the normal range. These results raised concerns for dRTA complicated by severe symptomatic hypokalemia.

**Table 1A:** Laboratory findings and blood analysis, including blood analysis, thyroid function tests, and parathyroid hormone levels. It provided diagnostic insights into the patient’s condition.

Parameter	Value	Normal range
Hemoglobin	139	115–150 g/L
Total leucocyte count	6.9	3.5–9.5 × 10^9^/L
Platelet count	291	125–350 × 10^9^/L
Erythrocyte sedimentation	Rate 49 mm fall in first hour	Up to 20 mm fall in first hour
C-reactive protein	0.36 mg/dL	<5 mg/dL
Blood urea	2.75	2.6–7.5 mmol/L
Serum potassium	3.09	3.5–5.3 mmol/L
Bicarbonate	15.5	22–29 mmol/L
Serum chlorine	110.1	99–110 mmol/L
Serum calcium	2.06	2.11–2.52 mmol/L
Serum phosphorus	0.62	0.97–1.62 mmol/L
Serum sodium	137.6	137–147 mmol/L
Anion gap	20.4	10–14 mmol/L
ABG analysis	pH 7.33 HCO3 14.4 pCO2 28.2 Anion gap 8	pH 7.35–7.45 HCO3 21–28 mmol/L pCO2 32–45 mm Hg Anion gap 7–16 mmol/L
Urine	pH 7.0	pH < 5.3 pH > 5.3 is suggestive of dRTA
Serum creatinine	60	41–73 μmol/L
Total bilirubin	6.1	3.42–20.5 μmol/L
Alanine transferase	21	<40 IU/L
Aspartate transferase	22	<40 IU/L
Total serum protein	89.4	65–85 g/L
Serum albumin	44.1	40–55 g/L
FT3	2.1	3.1–6.8 pmol/L
FT4	3.91	12–22 pmol/L
THS	>100	0.27–4.2 uIU/mL
PTH	32.18	15–65 pg/mL
Ophthalmological tests		
Tear breakup time	6 s	>10 s
Fluorescein eye stain	Positive	Negative

dRTA = distal renal tubular acidosis.

Further assessment with dual-emission X-ray absorptiometry indicated reduced bone mass in the lumbar spine and femoral neck, with a *Z*-score <−2.0, providing an explanation for the patient’s bone pain.

**Figure 1.**
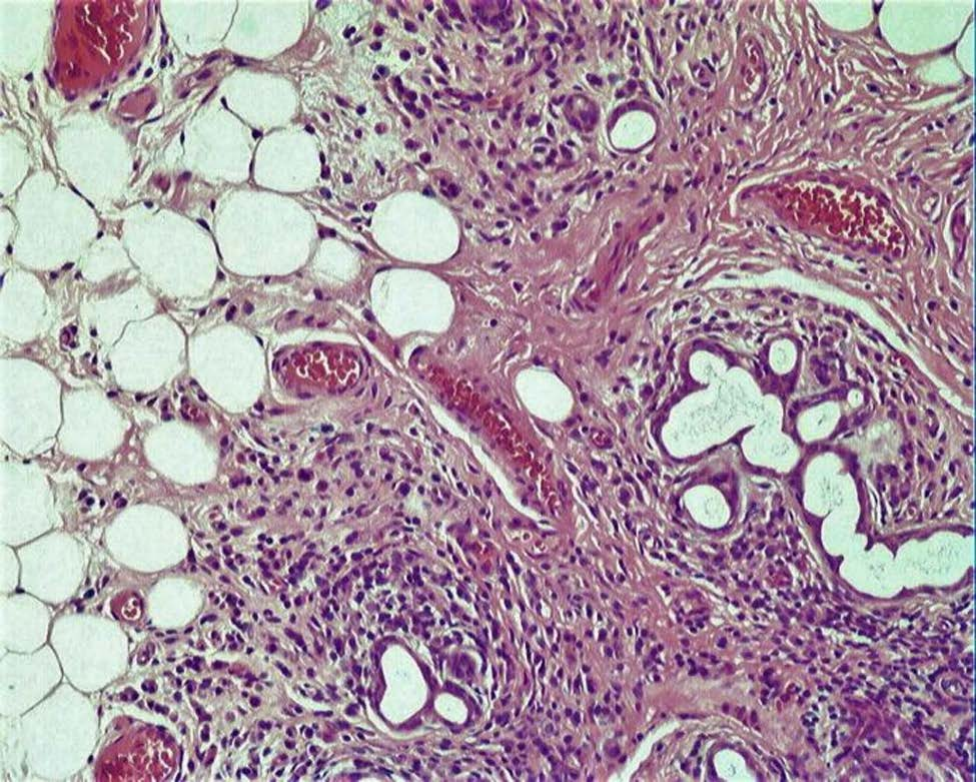
H&E staining of the patient’s labial gland tissue. It reveals significant interstitial infiltration by lymphocytes and plasma cells, indicative of local salivary gland atrophy. H&E = hematoxylin and eosin.

The patient was also found to suffer from chronic autoimmune thyroiditis and severe hypothyroidism, evidenced by low levels of thyroid hormones and elevated thyroid-stimulating hormone levels, exceeding the upper limit of the test range. Table 1B illustrates that several autoimmune antibodies were present at high levels, including antibodies against Sjögren’s syndrome antigens A and B, antinuclear antibodies, and rheumatoid factors, all of which are suggestive of SS. Despite the absence of dry eye symptoms, ophthalmological testing, including a 6-second fluorescein tear breakup time and a positive fluorescein eye stain test, indicated decreased tear film stability and tear secretion. A lower lip biopsy revealed chronic atrophic changes in the salivary glands with significant lymphocytic and plasma cell infiltration (Fig. 1). Integrating these findings, the patient was diagnosed with SS complicated by dRTA, hypothyroidism, and osteomalacia.

**Table 1B:** Autoimmune antibody detection results for the patient. It illustrates the presence and titers of various antibodies that are indicative of autoimmune activity, which supports the diagnosis of Sjögren’s syndrome and associated conditions.

Parameter	Value	Normal range
Antinuclear antibody (by indirect immunofluorescence)	Positive	Negative
Rheumatoid factor	53.2 IU/mL	0–20 IU/mL
Anti-CCP	Negative	Negative
Anti-SS-A	Positive	Negative
Anti-SS-B	Positive	Negative
Anti-Sm	Negative	Negative
HLA-B27	Negative	Negative
C3	0.79	0.9–1.8 g/L
C4	0.20	0.2–0.4 g/L
IgG	25.54	7–16 g/L
IgM	3.33	0.4–2.3 g/L
IgA	3.3	0.7–4.0 g/L
TRAB	<0.250	<1.5 IU/L
TPOAB	747.40	0–30 U/mL

CCP = cyclic citrullinated peptide, HLA = human leukocyte antigen, IgA = immunoglobulin A, IgM = immunoglobulin M, SS = Sjögren’s syndrome, TRAB = Thyrotrophin Receptor Antibody, TPOAB = thyroid peroxidase antibody.

### 2.2. Treatment and follow-up

The patient was administered a comprehensive treatment regimen that included potassium citrate granules at a dosage of 8.76 g/d, levothyroxine sodium tablets at 100 μg/d, calcitriol soft capsules at 0.25 μg/d, and calcium carbonate tablets at 600 mg/d. Concurrently, she received immunomodulatory therapy with hydroxychloroquine at a daily dose of 400 mg. To mitigate the risk of exacerbating bone mass loss, the patient opted for alendronate tablets at 70 mg once a week for osteoporosis treatment, deferring glucocorticoid therapy. Topical tear substitutes were also utilized to alleviate her dry eye symptoms.

After 2 weeks of inpatient treatment, her serum potassium levels stabilized within the range of 3.55 to 4.68 mmol/L, and there was a gradual improvement in thyroid hormone function, culminating in the resolution of her symptoms. She was discharged with a plan for close follow-up. Upon reevaluation as an inpatient 6 months later, her strength and weight had increased. Biochemical assays indicated that her thyroid hormone levels were maintained within the normal range, and the bone density of her lumbar spine and femoral neck had normalized (as detailed in Tables 2 and 3).

**Table 2:** Serum and hormone levels at 2-week and 6-month post follow-up interval. It demonstrates the patient’s biochemical response to therapy and normalization of key electrolytes and thyroid function indices.

Parameter	Normal range	2 weeks after treatment	6 months after treatment
Serum potassium	3.5–5.3 mmol/L	4.1	2.93
Bicarbonate	22–29 mmol/L	22.1	15.6
Serum chlorine	99–110 mmol/L	109.4	107.2
Serum calcium	2.11–2.52 mmol/L	2.15	2.29
Serum sodium	137–147 mmol/L	142.1	138.3
FT3	3.1–6.8 pmol/L	3.48	4.02
FT4	12–22 pmol/L	11.21	20.44
THS	0.27–4.2 uIU/mL	60.87	6.57

FT3 = freeTriiodothyronine, FT4 = freeThyroxine, THS = thyroid-stimulating hormone.

**Table 3:** Bone mineral density and *Z*-scores of the patient at baseline and after 6 months of treatment. It indicates improvements in bone health across various skeletal regions.

Scanning area	BMD	*Z*-value
Lumbar spine 1–4	0.799 g/cm^2^	‐2.3
The hip joint	0.602 g/cm^2^	‐2.5
The femoral neck	0.680 g/cm^2^	‐2.0
*6 months after treatment*		
Lumbar spine 1–4	1.055 g/cm^2^	‐0.1
The hip joint	0.862 g/cm^2^	‐0.3
The femoral neck	0.879 g/cm^2^	‐0.5

BMD = bone mineral density.

However, she continued to exhibit signs of renal injury, with mild hypokalemia (K+ levels between 2.93 and 3.4 mmol/L) and mild metabolic acidosis (HCO3‐ at 15.6 mmol/L). Notably, she experienced recurrent episodes of severe hypokalemia and acidosis upon discontinuation of the high-dose potassium supplementation. To prevent further renal damage, it was recommended that the patient initiate glucocorticoid treatment.

## 3. Discussion

This case exemplifies the intricacies of diagnosing and managing SS in a young woman, particularly when it presents with atypical symptoms such as hypokalemia paralysis. The patient’s clinical picture, characterized by muscle weakness, periodic paralysis, bone pain, and biochemical indicators of dRTA, underscored the complexity of SS and its systemic manifestations beyond the classic sicca symptoms.^[4]^ The diagnosis was further complicated by the concurrent development of osteomalacia, highlighting the importance of a thorough investigation in patients with SS who present with unexplained bone pain or muscle weakness.

Renal involvement in our patient, evidenced by dRTA, aligns with recent studies that have reported a varied prevalence of renal complications in SS, ranging from 0.3% to 27%.^[5]^ The pathophysiological link between SS and dRTA remains enigmatic but is thought to involve immune-mediated damage to the renal tubules, potentially triggered by the same autoantibodies that target salivary gland tissues.^[6,7]^ This shared autoimmune pathogenesis may explain the co-occurrence of sicca symptoms and renal tubular acidosis in some SS patients.

The therapeutic approach for our patient included potassium supplementation, thyroid hormone replacement, and immunomodulatory therapy with hydroxychloroquine, which has been suggested as a first-line treatment for inflammatory musculoskeletal pain associated with SS.^[8]^ The addition of alendronate for osteoporosis prevention was a prudent decision, given the risk of bone loss associated with dRTA and hypothyroidism.^[9]^ The eventual introduction of glucocorticoids, following the normalization of bone density, was based on evidence that supports their use in severe cases of SS with extraglandular manifestations.^[10]^

This case underscores the necessity for a multidisciplinary approach to the management of SS, particularly when patients present with rare complications. Early diagnosis and tailored treatment strategies are paramount to prevent disease progression and improve patient outcomes. Future research should focus on elucidating the mechanisms underlying the extraglandular manifestations of SS, including dRTA, to inform more targeted therapeutic interventions.

## 4. Conclusion

The case highlights the uncommon association of Sjögren’s syndrome with dRTA and osteomalacia, presenting as hypokalemic paralysis. The early diagnosis and a comprehensive treatment plan, including potassium supplementation and hormone replacement, led to symptom resolution and improved the patient’s quality of life. It reinforces the significance of recognizing atypical presentations of Sjögren’s syndrome to prevent complications and ensure better patient outcomes.

## Acknowledgments

The authors appreciate the patient’s consent to present this case.

## Author contributions

**Methodology:** Xiaokang Fang.

**Supervision:** Jie Ning.

**Writing – original draft:** Runtian Chen.

**Writing – review & editing:** Lihua Fang.
